# Evaluation of pyruvate decarboxylase‐negative *Saccharomyces cerevisiae* strains for the production of succinic acid

**DOI:** 10.1002/elsc.201900080

**Published:** 2019-08-29

**Authors:** Ahmed Zahoor, Felix T. F. Küttner, Lars M. Blank, Birgitta E. Ebert

**Affiliations:** ^1^ Institute of Applied Microbiology – iAMB Aachen Biology and Biotechnology – ABBt RWTH Aachen University Aachen Germany

**Keywords:** CRISPR‐Cas9, dicarboxylic acids, metabolic engineering, succinic acid, yeast

## Abstract

Dicarboxylic acids are important bio‐based building blocks, and *Saccharomyces cerevisiae* is postulated to be an advantageous host for their fermentative production. Here, we engineered a pyruvate decarboxylase‐negative *S. cerevisiae* strain for succinic acid production to exploit its promising properties, that is, lack of ethanol production and accumulation of the precursor pyruvate. The metabolic engineering steps included genomic integration of a biosynthesis pathway based on the reductive branch of the tricarboxylic acid cycle and a dicarboxylic acid transporter. Further modifications were the combined deletion of *GPD1* and *FUM1* and multi‐copy integration of the native *PYC2* gene, encoding a pyruvate carboxylase required to drain pyruvate into the synthesis pathway. The effect of increased redox cofactor supply was tested by modulating oxygen limitation and supplementing formate. The physiologic analysis of the differently engineered strains focused on elucidating metabolic bottlenecks. The data not only highlight the importance of a balanced activity of pathway enzymes and selective export systems but also shows the importance to find an optimal trade‐off between redox cofactor supply and energy availability in the form of ATP.

**Abbreviations**
OD_600_: optical density measured at 600 nm
PEP: phosphoenolpyruvate
REF: reference strain
SD: System Duetz
SF: shake flask
TCA: tricarboxylic acid

## 1 INTRODUCTION

Organic acids are industrially essential chemicals that are produced annually on a multi‐million‐ton scale for use in the food, pharmaceutical, and chemical industry. Many organic acids are metabolic intermediates, for example, of the tricarboxylic acid (TCA) cycle, and therefore can be naturally produced by a variety of microorganisms. For production in high amounts, however, metabolic engineering is typically required. *Saccharomyces cerevisiae* offers several advantages for the use as a host for organic acid production such as the ability to grow at low pH to enable the production of the protonated acid rather than the salt. The genetic toolbox is well established, and recent advances enable the efficient genomic insertion of large DNA fragments as well as deletions or mutations using the CRISPR‐Cas9 system 1, 2, 3. Simultaneous introduction of multiple genetic modifications (multiplexing) is also possible 4. Accordingly, *S. cerevisiae* has been used in a variety of studies to produce organic acids such as malic acid 5, fumaric acid 6, 7, and succinic acid 8, 9, 10.

Succinic acid has captured interest as a platform chemical, and there is plentiful research for its production using microbial cell factories 11, 12. Besides use as acid, its value as a precursor for bioplastics and bulk chemicals such as 1,4‐butanediol has attracted much attention 13, 14. Several industrial processes based on different host organisms have been patented and are used for bio‐based succinate production 15. On an industrial scale, *S. cerevisiae* strains are being used by Reverdia to produce 10,000 tons of succinate per year 11. The market size of succinic acid is expected to exceed 700,000 tons per annum by 2020, and the estimated cost of succinic acid made via fermentation ($0.55–$1.10 per kg) is comparable to the petrochemical route that uses maleic anhydride as raw material 11, 16.

**Figure 1:**
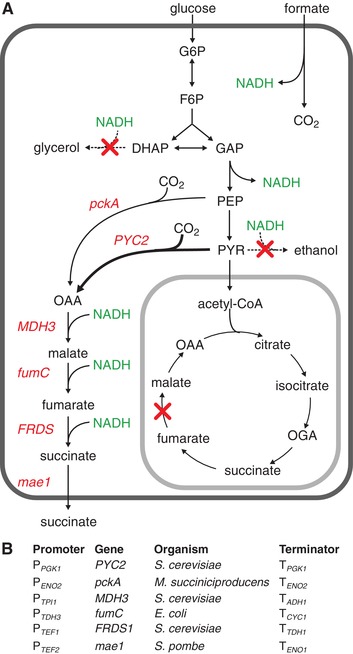
(A) A schematic depiction of the central carbon metabolism of *S. cerevisiae* and the metabolic engineering approach used in this study. The two carbon dioxide fixation reactions convert either PEP or pyruvate into oxaloacetate, which is then reduced in three further steps to succinate and finally exported outside of the cell by a heterologous transporter (Mae). (B) Description of the pathway genes and the donor organisms as well as the regulatory elements (all from *S. cerevisiae*) used for the construction of the integrated gene cassettes

Although *S. cerevisiae* has already been metabolically optimized for succinate production, the performance often suffers from high byproduct formation such as ethanol and glycerol 9, 10, 17, 18. So far, the genetic engineering strategies for succinate production with *S. cerevisiae* have been based on either the oxidative or the reductive TCA cycle route. For production via the oxidative route, Raab and colleagues deleted the succinate dehydrogenase genes to restrict oxidation of succinate to fumarate and the isocitrate dehydrogenase gene to enable succinate production via the glyoxylate shunt 10. This enabled extracellular accumulation of succinate (Table 1), but considerable amounts of ethanol, glycerol, and acids were also excreted into the production medium. Ito and colleagues built upon this approach and deleted five alcohol dehydrogenase genes (*ADH1*–*ADH5*) to eliminate the by‐product ethanol 18. This significantly reduced but did not cease ethanol formation while glycerol formation was also observed. Accumulation of these byproducts was also mentioned in another study that enabled succinate production via the deletion of mitochondrial malate transport 9. Further studies have engineered strains for succinate production via the reductive arm of the TCA cycle, which includes carbon dioxide fixation and offers higher product yield in comparison to the oxidative route 19. Yan and colleagues engineered the reductive pathway in a strain (TAM), which lacks the pyruvate decarboxylase genes and is incapable of ethanol production 20. This strain also had previously been evolved to be independent of the requirement of a C2 carbon source (e.g., acetate or ethanol) and does not exhibit glucose sensitivity, both of which are characteristics associated with pyruvate decarboxylase deletion mutants 21, 22. As the TAM strain also accumulates pyruvate, which can be reduced to succinate (Figure 1), it is a reasonable basis strain for succinate production. It is worth mentioning that there are several patents reporting very high titers for succinate with strains similarly engineered to those mentioned above and in this study. However, details such as growth characteristics, process set‐up, byproduct formation, and their concentrations are often missing.

**Table 1:** Comparison of succinate production parameters of engineered *S. cerevisiae* strains

Strain	Titer (g/L)	Yield (mol/mol)	Specific succinate production rate (mmol/g_CDW_/h)	Productivity (mg/L/h)	Reference
AH22ura3 ∆*sdh2*∆*sdh1*∆*idh1*∆*idp1*	3.6	0.11		0.02	10
PMCFfg	9.98	0.32		0.14	8
8D evolved + pICL1	0.9	0.03		–	53
S149sdh12 with *mae1*	0.41	0.03	0.19	0.01	18
∆*dic1*	0.23	0.02	0.4	–	9
FA1	1.96	0.17	0.06	0.04	This study

**PRACTICAL APPLICATION**

We explored the use of a pyruvate decarboxylase‐negative and pyruvate accumulating *Saccharomyces cerevisiae* strain for succinate production employing the reductive tricarboxylic acid (TCA) cycle. Compared with the oxidative branch of the TCA cycle and the glyoxylate shunt, this route allows a higher theoretical product yield on glucose. However, it is only superior when NADH availability required for the reductive steps is abundant. Here we show that NADH is limiting and that reducing competition for this crucial cofactor with the respiratory chain and increasing its formation by formate oxidation improves succinate synthesis. Moreover, an efficient drain of pyruvate into the reductive arm of the TCA requires high pyruvate carboxylase activity. This was achieved here by increasing the copy number of chromosomally integrated *PYC2* genes using a transposon‐sequence‐based strategy resulting in significantly boosted succinate production. These findings are of practical relevance for future efforts to engineer *S. cerevisiae* strains for dicarboxylic acid synthesis.

In this study, we exploited the potential of the TAM strain. We overexpressed (heterologous) genes for the carboxylation and further reduction of pyruvate to succinate and its export. In addition, we addressed redox cofactor supply and the enzymatic bottleneck of pyruvate carboxylation.

## 2 MATERIALS AND METHODS

### 2.1 Integration of the succinate production pathway

The strains and plasmids used in this study are listed in Table 2. For the implementation of the reductive succinate production pathway, six different plasmids carrying the genes *pckA*, *PYC2*, *MDH3*, *fumC*, *FRDS1*, and *mae1* were synthesized and the gene cassettes integrated into the yeast genome. Each gene cassette was equipped with a distinct promoter and terminator (Figure 1B). The promoter sequences consisted of at least 400 bp upstream, whereas terminator sequences were generally 225 bp downstream of the corresponding genes. The sequences of the heterologous genes were codon‐optimized for expression in *S. cerevisiae*. All sequences are provided in the Supporting Information. The promoters were selected for constitutive expression in *S. cerevisiae* based on previous studies 23, 24. Except for *PYC2* (which was cloned into pCfb255), all cassettes were synthesized and sequenced by Thermo Fisher Scientific. From the plasmids, each cassette was amplified using primers carrying a 60 bp homologous sequence with the amplification primer of the cassette it was to be recombined with 1. The first and last cassettes additionally carried sequences homologous to the X2 insertion site on the genome as well as the selection marker (*KlURA3*) 25. The amplified gene cassettes were transformed into yeast cells, and genomic integration was confirmed by colony PCR 26.

**Table 2:** List of strains and plasmids used in this study

Strain	Description	Reference
REF	*S. cerevisiae* CEN.PK 111–61A. MATα *ura3‐52 leu2‐112 his3‐Δ1*	Euroscarf
REF‐5	REF with genomic insertion of PYC2‐PCKA‐MDH3‐FUMC‐FRDS1 pathway	This study
REF‐6	REF with genomic insertion of PYC2‐PCKA‐MDH3‐FUMC‐FRDS1‐MAE1 pathway	This study
TAM	*S. cerevisiae* MATa *pdc1(−6,−2)::loxP pdc5(−6,−2)::loxP pdc6(−6,−2)::loxP ura3‐52*	20
TAM‐5	TAM with genomic insertion of PYC2‐PCKA‐MDH3‐FUMC‐FRDS1 pathway	This study
TAM‐6	TAM with genomic insertion of PYC2‐PCKA‐MDH3‐FUMC‐FRDS1‐MAE pathway	This study
TAM‐6 ∆*gpd1*	TAM‐6 with *GPD1* deletion	This study
TAM‐6 ∆fum*1*	TAM‐6 with *FUM1* deletion	This study
TAM‐6 ∆*gpd1 ∆fum1*	TAM‐6 with *GPD1* and *FUM1* double deletion	This study
FA	TAM‐6 *gpd1*∆ *fum1∆ leu2∆*	This study
FA1	FA with multiple genomic insertion of *PYC2* gene‐cassette	This study

**Plasmid**		
pCfb255	Easyclone integrative vector with loxP flanked URA3 marker. Amp^r^	25
pCfb255‐*PYC2*	pCfb255 carrying the *PYC2* cassette. Amp^r^	This study
pMK‐RQ‐*pckA*	Plasmid carrying the *pckA* cassette. Kan^r^	This study
pMA‐RQ‐*MDH3*	Plasmid carrying the *MDH3* cassette. Amp^r^	This study
pMK‐RQ‐*fumC*	Plasmid carrying the *fumC* cassette. Kan^r^	This study
pMK‐RQ‐*mae1*	Plasmid carrying the *mae1* cassette. Kan^r^	This study
pMA‐*FRDS1*	Plasmid carrying the *FRDS1* cassette. Amp^r^	This study
pCfB2312	CRISPR‐Cas9 plasmid carrying the Cas9 endonuclease. KanMX^r^	34
pCfB3496	CRISPR‐Cas9 plasmid for cloning the gRNA. Hph^r^	3
pCfB3496‐*GPD1*	CRISPR‐Cas9 plasmid carrying the gRNA for *GPD1*	This study
pCfB3496‐*LEU2*	CRISPR‐Cas9 plasmid carrying the gRNA for *LEU2*	This study
pCfB2991	pTY1Cons2‐Kl*LEU2*‐deg.	46
p2991‐*PYC2*‐Ty	Plasmid carrying the *PYC2* gene‐cassette with overhangs homologous to the Ty1Cons2 sites in the genome and a weakened *LEU2* marker. Amp^r^	This study

### 2.2 CRISPR/Cas9 mediated deletion of *GPD1* and *LEU2*

A CRISPR/Cas9 toolset was used for the deletion of *GPD1* and *LEU2* 3. Although *GPD1* was deleted to reduce the loss of carbon and reduced cofactors in the glycerol formation pathway, *LEU* deficiency was required for the multi‐copy integration of *PYC2*, for which *LEU2* was used as a selection marker. The CRISPR/Cas9 toolset consisted of two plasmids: pCfB2312 carried the Cas9 endonuclease and was transformed in combination with pCfB3496, which carried the gRNA for the desired locus and a repair oligo complementary to sequences up‐ and downstream of the locus to be deleted. gRNA and repair oligos were designed using the web‐based tool yeastriction 4. The colonies obtained after transformation were tested for successful deletion using colony PCR. Plasmid curing was achieved by streaking the clones on YEP agar plates without antibiotics. Plasmid loss was confirmed by the inability of clones to grow on medium with antibiotics.

### 2.3 KanMX resistance cassette mediated *FUM1* deletion

For deletion of *FUM1*, a KanMX resistance cassette was amplified with 600 bp overhangs containing sequences homologous to the upstream and downstream regions of the *FUM1* gene 27. This DNA fragment was transformed into yeast cells, and deletion of *FUM1* was confirmed by the PCR products obtained using a set of primers binding upstream of the *FUM1* locus and within the KanMX cassette and a PCR reaction using a primer set binding upstream of the *FUM1* locus and within the *FUM1* gene.

### 2.4 Cultivation conditions

The strains were routinely grown at 30°C either in 500 mL shake flasks (SFs) filled with 50 mL medium or in 24 deep‐well plates (Enzyscreen, System Duetz^®^) with a filling volume of 1.5 mL per well. A shaking speed of 200 rpm in a rotary shaker (Infors Multitron, Switzerland) with 25 mm amplitude was used for aerobic cultivations. The shaking speed was reduced to 90 rpm for microaerobic growth.

Overnight cultures were made by inoculating cells in YEP medium (1% yeast extract, 1% peptone, 0.5% sodium chloride, and 2% glucose). For production experiments, the overnight culture was centrifuged, washed, and re‐suspended in mineral salt medium to a starting OD_600_ (optical density measured at 600 nm) of 1. The composition of the mineral salt medium was as follows: 3 g/L KH_2_PO_4_, 0.5 g/L MgSO_4_·7H_2_O, 0.4 g/L urea, 6.6 g/L K_2_SO_4_, 0.015 g/L EDTA, 0.0045 g/L CaCl_2_·H_2_0, 0.0045 g/L ZnSO_4_·7H_2_0, 0.003 g/L FeSO_4_·7H_2_0, 0.0003 g/L CuSO_4_·4H_2_O, 0.001 g/L MnCl_2_·4H_2_O, 0.0004 g/L Na_2_MoO_4_·2H_2_O, 0.0003 g/L CoCl_2_·6H_2_O, 0.001 g/L H_3_BO_3_, and 0.0001 g/L KI. Vitamins were added at a final concentration of: 0.25 g/L myo‐inositol, 0.01 g/L nicotinic acid, 0.01 g/L pyridoxine‐HCl, 0.01 g/L thiamin‐HCl, 0.0005 g/L biotin, 0.01 g/L calcium pantothenate, and 0.002 g/L *p*‐aminobenzoic acid 8. The medium was routinely buffered with 30 g/L of calcium carbonate, and 5% glucose was used as a substrate.

### 2.5 Analytical methods

All SF cultivation experiments were performed in triplicates. The data shown are the arithmetic mean of the triplicates with the corresponding standard deviations. When calcium carbonate (CaCO_3_) was used as a buffer, the samples were treated with concentrated hydrochloric acid (HCl) prior to measuring the optical density (OD_600_) or running HPLC analysis. One milliliter of culture was mixed with 400 µL of concentrated HCl and 600 µL water to dissolve the CaCO_3_ completely. The OD_600_ was measured using an Ultrospec 10 cell density meter (Amersham Biosciences, UK) and converted to biomass concentrations using a conversion factor of 0.21 g_CDW_/L per OD_600_ previously determined by Czarnotta et al. 28.

HPLC was used to quantify extracellular metabolites from acidified supernatant of culture samples. The HPLC system (Beckman Coulter, Brea) was equipped with a UV detector (Beckman Coulter, Brea) set to a wavelength of 210 nm, and a refractive index detector operated at 40°C (Knauer, Germany). Analyte separation was achieved with an Organic Acid Resin column (CS Chromatographie, Germany) eluted with 30 mM sulfuric acid containing 1% (v/v) acetonitrile using an isocratic flow of 0.6 mL/min.

## 3 RESULTS AND DISCUSSION

### 3.1 Design of the recombinant succinate synthesis pathway

We expressed the genes for the anaplerotic enzymes pyruvate carboxylase and phosphoenolpyruvate carboxykinase, the genes for the reductive TCA cycle enzymes malate dehydrogenase, fumarase, and fumarate reductase as well as a gene encoding a malic acid transporter to enable succinate production and export. One of the challenges was enabling cytosolic expression of all these reactions because not all of them exist naturally in *S. cerevisiae*, proceed in the reversed direction or are localized to another compartment. Hence, genes were expressed heterologously to have a complete pathway, and native genes were modified to be localized in the cytosol instead of in the mitochondrion.

Phosphoenolpyruvate (PEP) carboxykinase from the natural succinate producer *Mannheimia succiniciproducens* (encoded by *pckA*) (EC 4.1.1.49) was chosen for the conversion of PEP to oxaloacetate. The PEP carboxykinase has been shown to be the most crucial carbon dioxide‐fixing reaction for succinate production in this strain 29. Additionally, its expression in a eukaryotic host has been documented to improve succinate formation 30. The second anaplerotic reaction was the pyruvate carboxylase (EC 6.4.1.1) encoded by *S. cerevisiae*’s native *PYC2* gene. Because the TAM strain accumulates pyruvate, overexpression of the native pyruvate carboxylase should enable pyruvate conversion to oxaloacetate and eventually to succinate with the downstream pathway enzymes. *S. cerevisiae* possesses three malate dehydrogenases (EC 1.1.1.37) responsible for NADH‐dependent OAA reduction to malate. *MDH3* encoding the peroxisomal isozyme was chosen as the cytosolic form (*Mdh2*) is repressed by glucose 31. Mdh3 was relocalized to the cytosol by removing the peroxisomal targeting sequence as previously reported by Yan et al. 8. Fumarase (EC 4.2.1.2) catalyzes the reversible hydration/dehydration of fumarate to malate. In *S. cerevisiae*, the fumarase is known to function in the direction of fumarate hydration to malate 32 and is located both in the cytoplasm and the mitochondria 33. Cytoplasmic conversion of malate to fumarate was achieved by expression of the *Escherichia coli* fumarase gene *fumC* 34. The last step in the pathway is catalyzed by the native cytosolic fumarate reductase, encoded by *FRDS1*, which was overexpressed. Export of succinate was enabled by heterologous expression of *mae1*, encoding a *Schizosaccharomyces pombe* malic acid transporter, known to reversible transport malate as well as similar dicarboxylic acids such as succinate and fumarate 18, 35.

### 3.2 Chromosomal expression of the heterologous pathways in the wild‐type and TAM strain

**Figure 2:**
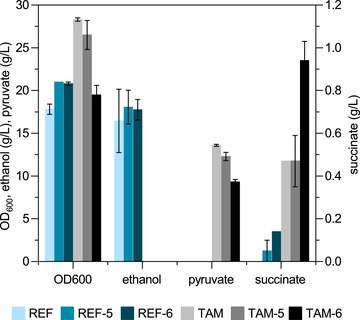
Comparison of the reference and TAM strains engineered for succinate production. The strains were cultivated in mineral salt medium with 5% glucose as substrate and supplemented with 22 mm formate under microaerobic conditions (shaking frequency 90 rpm). Data presented are the mean and standard deviation of triplicate samples taken at 144 h

The above‐described pathway was integrated into the genome of the *S. cerevisiae* CEN.PK reference strain (designated as REF) and the pyruvate decarboxylase mutant TAM either completely (six‐step pathway) or excluding the Mae1 transporter (five‐step pathway) 20. The strains were grown in a mineral salt medium under microaerobic conditions by setting the shaking frequency to 90 rpm. Growth was followed by measuring OD_600,_ and culture supernatants were analyzed for organic acid production. The TAM strain reached a higher OD_600_ (28 ± 0) compared to the REF (18 ± 1). Integration of the five‐step pathway into the TAM strain (TAM‐5) did not affect the final biomass concentration, but the six‐step pathway reduced it by approximately 30% to an OD_600_ of 20 (Figure 2). Conversely, expression of both the five‐ and six‐step (REF‐5 and REF‐6) pathways had a positive effect on biomass formation of the wild‐type strain and the final OD_600_ increased by almost 18% and 17%, respectively. Interestingly, the TAM strain was capable of producing succinate even without expressing the pathway genes and accumulated approximately 0.5 g/L succinate. The same titer was observed with the five‐step pathway. In contrast, succinate production with the wildtype was only observed after integration of the succinate synthesis pathways. The expression of the *mae1* gene was critical in both strain backgrounds and led to an almost three times higher succinate titer in the wildtype (REF‐6) and a twofold increase in the TAM strain (TAM‐6) compared with the strains carrying the succinate synthesis pathway but lacking the transporter. In several other studies, introduction of a heterologous transporter function has been shown to be critical for the extracellular accumulation of several organic acids including succinate 5, 18, 36

The TAM strain did not produce ethanol, the main by‐product formed by the wild‐type strain in concentrations as high as 18 g/L, but similar amounts of pyruvate. Although pyruvate titers decreased in the engineered strains, the continuing accumulation of this precursor indicated a limitation in the downstream succinate pathway. Nevertheless, the higher product titer and the absent formation of ethanol suggested the TAM strain to be a better chassis for succinate production compared with the reference strain.

### 3.3 Effect of *GPD1* and *FUM1* deletion on product titers

Glycerol was the second major by‐product produced by the TAM strains and constituted a considerable carbon loss (3–6% w/w, Table 3). Furthermore, it is known that the fumarase of *S. cerevisiae* functions primarily in the oxidative direction, whereas for the pathway designed in this study a function in the reverse direction is desired (Figure 1) 32. Accordingly, we aimed to delete these activities, singly and in combination, in the TAM strain carrying the six‐step pathway (TAM‐6). The generated strains were grown in mineral salt medium, and their growth, as well as organic acid production, was analyzed. Deletion of glycerol phosphate dehydrogenase encoding *GPD1* did not significantly impact the biomass formation, whereas the deletion of fumarase reduced it by approximately 14%; the double deletion mutant also showed a similar growth deficiency (Table 3). Single as well as double deletion of *GPD1* and *FUM1* resulted in an increased malate and pyruvate titer while the succinate titer increased only in the double knockout mutant and a maximal titer of 2.2 g/L was determined, an almost 2.5‐fold improvement compared with the TAM‐6 strain. Intriguingly, the glycerol titer increased in the strains deficient in *FUM1* and was not completely abolished by *GPD1* deletion (Table 3). Residual glycerol formation was also observed by Yan et al. and is explained by the *GPD1* paralog *GPD2* 8. The additional deletion of *GPD2* was, however, not considered as it results in an osmosensitive phenotype and renders cells incapable of anaerobic growth 37.

**Table 3:** Growth, organic acid, and glycerol formation by the engineered strains. Strains were cultivated in SD deep‐well plate and shaken at 90 rpm. Mineral salt medium with 50 g/L glucose and 22 mM formate was used. The data presented are the mean and standard deviation of triplicate samples taken after 144 h

		g/L			
Strain	OD_600_	Malate	Pyruvate	Succinate	Glycerol
TAM	28.3 ± 0.2	0.3 ± 0.0	13.6 ± 0.1	0.5 ± 0.0	3.1 ± 0.5
TAM‐6	19.5 ± 1.1	0.4 ± 0.0	9.3 ± 0.3	0.9 ± 0.1	1.4 ± 0.7
TAM‐6 *gpd1*∆	20.5 ± 2.5	0.8 ± 0.0	13.9 ± 1.7	0.8 ± 0.1	2.2 ± 0.8
TAM‐6 *fum1*∆	16.7 ± 0.9	0.7 ± 0.0	14.2 ± 5.1	1.1 ± 0.1	6.7 ± 0.7
TAM‐6 *gpd1*∆ *fum1*∆	15.3 ± 1.5	1.1 ± 0.0	18.2 ± 0.4	2.2 ± 0.1	3.8 ± 0.3

The microaerobic cultivation conditions applied in these experiments favored the production of the reduced byproduct glycerol 38. Indeed, shaking at a higher frequency (200 rpm) resulted in cessation of glycerol synthesis in the TAM‐6 *gpd1*∆ *fum1*∆ strain but also stopped succinate production (data not shown). Apparently, the introduced succinic acid pathway could not compete with the native glycerol synthesis pathways or respiratory chain for the reduced cofactor NADH, or the (recombinant) enzyme activity was limiting the reduction of oxaloacetate to succinate. Weakening *GPD2* expression, for example, by promoter exchange, might be helpful to minimize glycerol production and redirect flux toward succinic acid production while avoiding the negative effects associated with complete glycerol dehydrogenase deficiency.

### 3.4 Effect of oxygen limitation and formate supplementation on succinate production by TAM‐6 *gpd1*∆ *fum1*∆

**Figure 3:**
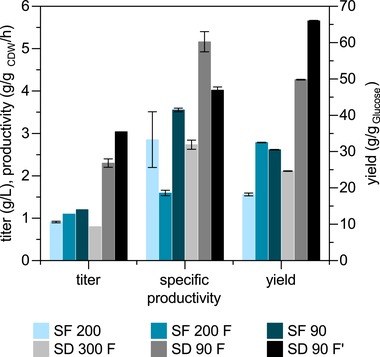
Effect of formate supplementation and oxygen limitation on succinate production in the TAM‐6 *gpd1*∆ *fum1*∆ strain in SFs and SD. The numeric value after the SF/SD depicts the shaking frequency (in rpm), F stands for formate supplementation. F’ represents the experiment with higher starting biomass concentration (OD_600_ of 10). The data are the mean of triplicate experiments; error bars show the standard deviation

To further test if NADH availability limited succinate production in the best producing strain, TAM‐6 *gpd1*∆ *fum1*∆, we modulated the oxygen transfer rate by changing the shaking frequency and adding formate (22 mM) to the medium. While O_2_ limitation reduces competition for NADH oxidation with the NADH dehydrogenases of the respiratory chain, the oxidation of formate to CO_2_ results in NADH formation and hence increased redox cofactor supply 39. As the two formate dehydrogenase genes (*FDH1* and *FDH2*) in *S. cerevisiae* are induced by formate, no additional strain engineering was done to test the impact of adding this additional energy source 40. The experiments were carried out in SFs or System Duetz (SD) 24‐well microplates, which enabled a higher throughput compared to flasks. The strain was grown in mineral salt medium with 5% glucose, and the succinate production under the above‐mentioned conditions was studied (Figure 3). In SFs, formate supplementation improved succinate titer (+21%) as well as yield on glucose (+78%). Conversely, growth was slower, resulting in decreased productivity. Oxygen limitation, realized by shaking at 90 rpm, had a positive influence on succinate production and increased titer, yield, as well as productivity by +32%, +68%, and +25%, respectively. Oxygen limitation in SD cultivations resulted in a more pronounced improvement with succinate titer, productivity, and yield increased by +150%, +80%, and +170%, respectively. Use of a higher starting OD_600_ of 10 (SD 90 F’, Figure 3) yielded the highest succinate titer (3 g/L) in this study and further improved yield (+33%), whereas the specific productivity was reduced (–22%).

The different performance in the two cultivation settings might be explained by differences in the oxygen transfer rate, which according to Meier et al. is much lower in SFs at the set frequency of 90 rpm, potentially too low to sustain optimal ATP formation and succinate production 41. A more in‐depth study of the dependence of succinate synthesis on oxygen availability might be valuable to improve production further.

A similar positive impact of NADH availability on organic acid biosynthesis has been reported previously for *S. cerevisiae* as well as *Yarrowia lipolytica* 42, 43. Also, it has been shown that under anaerobic conditions, the intracellular concentration of succinate and other TCA cycle intermediates is higher compared to their concentration under aerobic growth 44. Lifting redox cofactor limitations by oxidation of formate has also successfully been applied for succinate synthesis in *Corynebacterium glutamicum*. Heterologous expression of formate dehydrogenase in the engineered strain improved succinate yield by more than 20% 45. Although formate addition resulted in increased succinate synthesis in our studies as well, we also observed glycerol formation in cultures grown at reduced shaking frequency and with formate supplementation (data not shown). The secretion of this reduced by‐product indicates that under this condition the reductive succinate synthesis pathway can either not fully oxidize the NADH due to enzymatic limitations or not efficiently compete with the residual glycerol dehydrogenase activity.

### 3.5 Multiple genomic integrations of the pyruvate carboxylase (*PYC2*) cassette

The engineered yeast strain still accumulated a significant amount of pyruvate also under optimized conditions (Table 3) further supporting the hypothesized enzymatic bottleneck in the succinate biosynthesis pathway. To resolve this potential limitation, multiple copies of the pyruvate carboxylase gene were integrated into the genome of strain TAM‐6 *gpd1*∆ *fum1*∆ by targeting Ty retrotransposon elements 46. As many as 331 Ty insertions have been identified, spread across the *S. cerevisiae* genome of strain S288c 47. These sites are attractive for multiple genomic integrations of heterologous DNA (Figure 1), which has been used to tackle metabolic bottlenecks and improve production 11, 48, 49. We did not quantify the number of PYC2 copies integrated into the genome but assume similar copy numbers as reported by Borodina and coworkers 48, 50, that is, in the range of 2–4 copies.

**Figure 4:**
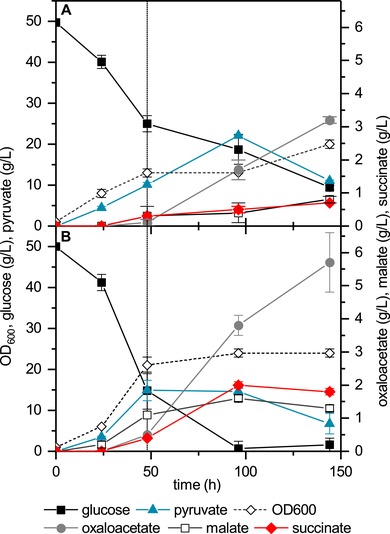
Two‐stage cultivation of the strains TAM‐6 *gpd1*∆ *fum1*∆ (A) and FA1 (B). Cultivation was carried out at 300 rpm for the first 48 h (dotted line) and then switched to 90 rpm to enable optimal organic acid production

Multi‐copy integration of the *PYC* gene resulted in growth deficiency under oxygen‐limiting conditions (see the Supporting Information). We hypothesized that this growth defect was caused by a limited supply of ATP for cellular processes under those microaerobic conditions caused by increased consumption of ATP for pyruvate carboxylation. Indeed, shaking at higher rpm relieved the growth suppression, and the strain (FA1) grew as well as the parental strain (TAM‐6 *gpd1*∆ *fum1*∆) and importantly produced three times less pyruvate. To achieve a compromise between growth deficiency of FA1 and optimal succinate production under oxygen‐limiting conditions, a two‐stage production approach was followed. During the first stage, the strain was grown at 300 rpm for 48 h to enable efficient biomass formation; afterward, the shaking speed was reduced to 90 rpm for efficient succinate production (Figure 4). For comparison, strain TAM‐6 *gpd1*∆ *fum1*∆ was also cultivated under the same conditions and growth as well as organic acid production were regularly monitored (Figure 4). Both strains merely produced carboxylic acid during the aerobic growth phase, but instead accumulated pyruvate, which was consumed after glucose depletion and converted to succinate, oxaloacetate, and malate. The FA1 stain produced almost 40% less pyruvate and reached a more than 2.5 times higher succinate titer (2 g/L) and specific productivity (maximum of 7 mg/g_CDW_/h in the initial nongrowing production stage) than its parental strain cultivated under the same conditions. Simultaneously an increase of almost 80% and 62% was noted for the oxaloacetate and malate titer compared to the parental strain.

Compared to another engineered TAM strain grown under similar conditions 8, FA1 produced nearly three times less pyruvate. This highlights the effectiveness of the multicopy integration of the pyruvate carboxylase cassette to channel more pyruvate into the reductive arm of the TCA cycle. However, the secretion of the succinate precursors oxaloacetate and malate points to additional enzymatic limitations as well as the high affinity of the Mae1 exporter for malate.

In addition to strain engineering, this study also emphasizes the importance of process engineering. Improved production of succinate and utilization of accumulated pyruvate was achieved when a two‐stage concept was applied, in which an initial aerobic growth phase was followed by an oxygen‐limited production phase. Such a decoupling of growth and production has also been shown to be of relevance for high‐level succinate production via the reductive arm of the TCA cycle in engineered *E. coli* strains 51, 52.

Vemuri and colleagues showed that for the dual‐phase fermentation, the transition time of the culture from growth to production phase has a significant effect on succinate accumulation and that highest succinate productivity is achieved when the aerobic phase takes place at a medium oxygen‐transfer rate 52. Although out of scope of this study, a detailed bioprocess optimization including the shift between the two stages will be critical to further develop the *S. cerevisiae‐*based process toward high titers, yields, and rates.

## 4 CONCLUDING REMARKS

We have elucidated potential metabolic bottlenecks in engineered *S. cerevisiae* limiting efficient succinate production and report strategies to overcome those drawbacks. Identified enzymatic and redox cofactor limitations were addressed by multiple genomic integrations of pyruvate carboxylase encoding *PYC2* and by reducing the oxygen transfer and hence the activity of competing NADH dehydrogenases as well as by addition of formate as an auxiliary energy source. Separation of growth and production proved useful to sustain the required energy demands for both processes. A transporter with higher specificity for succinate would be on our wishlist when working on the next generation strains. This study contributes to the ongoing discussion on the production of organic acids in yeasts and the challenges associated with redox cofactor balancing.

## CONFLICT OF INTEREST

The authors have declared no conflict of interest.

## Supporting information

Supporting Information
