# Supplementary material for: Evaluation of pyruvate decarboxylase‐negative Saccharomyces cerevisiae strains for the production of succinic acid
Source: Eng Life Sci. 2019 Aug 29;19(10):711–20. doi: 10.1002/elsc.201900080 (PMC6999389; doi:10.1002/elsc.201900080)
Supplement: Supplementary file 1 — Supporting Information [file ELSC-19-711-s001.pdf]

Supplementary file

Evaluation of pyruvate-decarboxylase-negative *Saccharomyces cerevisiae* strains for the production of succinic acid

Ahmed Zahoor, Felix T. F. Küttner, Birgitta E. Ebert, Lars M. Blank

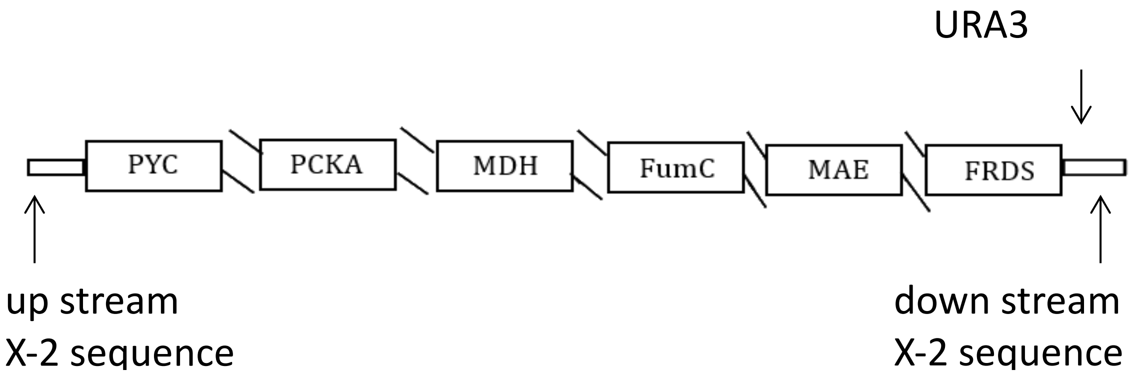

Figure S1. Diagram representing the order of the gene cassettes integrated into the genome and their components. Each cassette consists of a promoter-gene-terminator combination. The PYC gene-cassette is the first one in sequence and additionally has the sequence homologous to the X2-site in the genome on its 5' end. The FRD cassette carries the URA3 marker to complement the uracil auxotrophy as well as the sequence homologous to the X2-site in the genome on its 3' end. Each of these cassettes was cloned on a vector and amplified with primers having 60 bp overhangs to the primer used to amplify the neighboring cassette. The amplified cassettes were transformed into yeast, and genomic integration confirmed via PCR using primers pairs that bind to different cassettes.

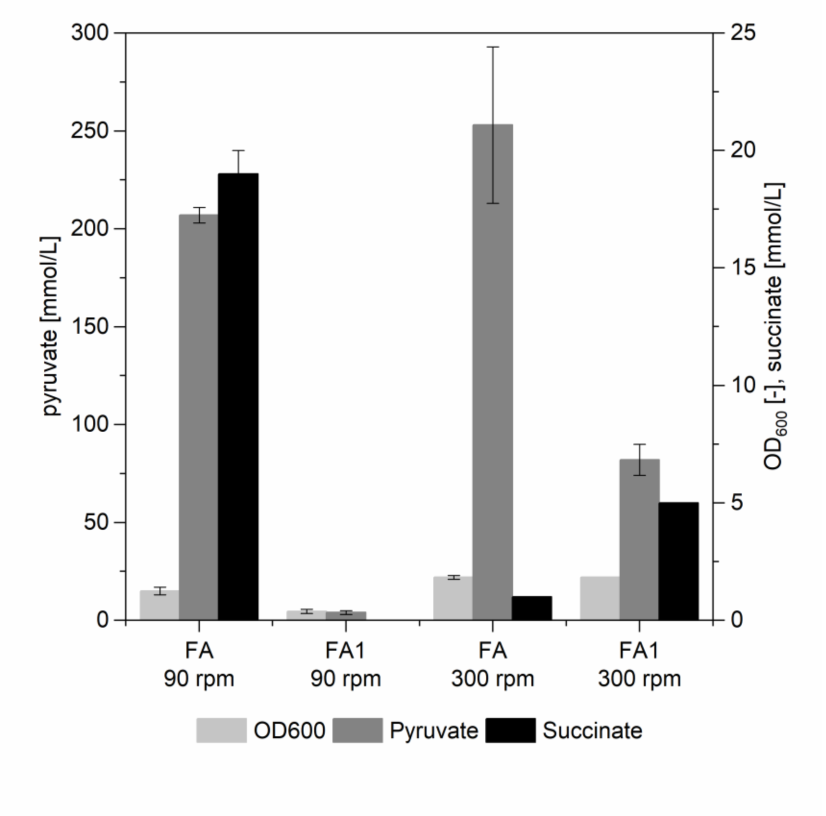

Figure S2. The FA1 strain was generated by a multicopy integration of the pyruvate carboxylase encoding gene *PYC2* into the genome of *S. cerevisiae* TAM-6 *gpd1*Δ *fum1*Δ (FA) strain. The growth of this strain, however, was strongly affected under oxygen limiting conditions optimal for succinate production. The growth defect was alleviated by growth at 300 rpm at which this strain grew almost as well as the parental strain followed by a microaerobic production stage. Importantly, this resulted in a substantial reduction in pyruvate titer and improvement in succinate production.

Table S1. List of primers used in this study

**Primer**	**Sequence**	**Description**
pUSER-PYC2_F	CGTCTATGAGGAGACTGTTAGTTG	Primer to amplify the *pyc*cassette
pUSER-PYC2_R	GTGCCTATTGATGATCTGGCGGAATGTCTGCCGTGCCATAGCCATGCCTTCACATATAGTACATAGAAATATCGAATGGGA	Primer to amplify the *pyc*cassette
PCKA_F	ACTATATGTGAAGGCATGGCTATGGCACGGCAGACATTCCGCCAGATCATCAATAGGCACGTGTCGACGCTGCGGGTATAGA	Primer to amplify the *pcka*cassette
PCKA_R	GTTGAACATTCTTAGGCTGGTCGAATCATTTAGACACGGGCATCGTCCTCTCGAAAGGTGATTTTTCAAACTGCAAATTCAAG	Primer to amplify the *pcka*cassette
MDH3_F	CACCTTTCGAGAGGACGATGCCCGTGTCTAAATGATTCGACCAGCCTAAGAATGTTCAACTATATCTAGGAACCCATCAGGT	Primer to amplify the *mdh3*cassette
MDH3_R	CTAGCGTGTCCTCGCATAGTTCTTAGATTGTCGCTACGGCATATACGATCCGTGAGACGTGAAATGGGGAGCGATTTGCAG	Primer to amplify the *mdh3*cassette
FUMC_F	ACGTCTCACGGATCGTATATGCCGTAGCGACAATCTAAGAACTATGCGAGGACACGCTAGTTAGTCAAAAAATTAGCCTT	Primer to amplify the *fumc* cassette
FUMC_R	AATCACTCTCCATACAGGGTTTCATACATTTCTCCACGGGACCCACAGTCGTAGATGCGTCTTCGAGCGTCCCAAAACCTTC	Primer to amplify the *fumc* cassette
MAE_F	ACGCATCTACGACTGTGGGTCCCGTGGAGAAATGTATGAAACCCTGTATGGAGAGTGATTGGGGCCGTATACTTACATATAG	Primer to amplify the *mae1* cassette
MAE_R	TGCCGAACTTTCCCTGTATGAAGCGATCTGACCAATCCTTTGCCGTAGTTTCAACGTATGATACATGGGTGACCAAAAGAGC	Primer to amplify the *mae1* cassette
FRDS_F	CATACGTTGAAACTACGGCAAAGGATTGGTCAGATCGCTTCATACAGGGAAAGTTCGGCAATAGCTTCAAAATGTTTCTAC	Primer to amplify the *frds* cassette
FRDS_R	CTCGCCAAGGCATTACCATCCCA	Primer to amplify the *frds* cassette
gpd1-gRNA_F	GTGGTTGCCGAAAATTGTAAGTTTTAGAGCTAGAAATAGCAAG	Primer with *gpd1* gRNA as overhang. Used to amplify the plasmid pCfb3496
gRNA_R	GATCATTTATCTTTCACTGCGGAGAAG	Primer to amplify and construct pCfb3496 carrying *gpd1* gRNA
gpd1-del-conf_F	AGGCCAAGACAGGGTCAATG	Primer to confirm *gpd1* deletion
gpd1-del-conf_R	TACATACGGACGCCAGATGC	Primer to confirm *gpd1* deletion
leu2-gRNA_F	TGATTATAATACCATTTAGGGTTTTAGAGCTAGAAATAGCAAG	Primer to amplify and construct pCfb3496 carrying *leu2* gRNA. As reverse primer 'gRNA_R' was used

Pyc-Ty_F	TGTTGGAATAAAAATCAACTATC	Primer to amplify the pyc gene cassette with up and downstream retrotransposon sequences
Pyc-Ty_R	TGAGAAATATGTGAATTTTGAGATAATTG	Primer to amplify the pyc gene cassette with up and downstream retrotransposon sequences

Sequences of the integrated gene cassettes

1. PYC (X2 sequence – Promoter pGK1 – PYC2 – Terminator PGK1)

CGTCTATGAGGAGACTGTTAGTTGGATATCAGTAATGAGACGAAAAAGCTCGAAATGAATGG
ATATATTCTTTTTGCTACTGGCAACTGTTGAATATTTAATGTTAAAACAAACTAACTGAGGTA
TATTCGTATCTGTATGTACACATATACTATATACAGGAAAAGATAAGCAAGAGAGAGGATAT
CAACTACGAGAGCGATCGATTATATATCAAAAGCTGTCCGCTTTGCCACCCATAATCGGCGC
TTAGTTTCGGAGTTCAATCATAATTCTACCACCTTACACTCAACTTACTCTTTAACTCCTATAG
TATAATATCGCCACTGACCCCATATTAAAAAATTTTTTTGCTCGATCTTCTATCCTCTTTAGGT
TAATTGTCGCTGTTATTGTCTAGATTTTTTCTCGGAGATGGCGCATCTATTTGCCGTCAAAAG
ATCCTCTCATACCATATTAAGTAAATTGCCTCCATTTCTTTTTCCTCGGGCAGAGAAACTCGC
AGGCAACTTGCTCTCGAAGTGGTCACGTATTAAGTCCTCAGCGAGCTCGCATGGAATGCGTC
AGGCATGAACGCATCACAGACAAAATCTTCTTGACAAACGTCACAATTGATCCCTCCCCATC
CGTTATCACAATGACAGGTGTCATTTTGTGCTCTTATGGGACGATCCTTATTACCGCTTTCATC
CGGTGATAGACCGCCACAGAGGGGCAGAGAGCAATCATCACCTGCAAACCCTTCTATACACT
CACATCTACCAGTGTACGAATTGCATTCAGAAAACTGTTTGCATTCAAAAATAGGTAGCATA
CAATTAAAACATGGCGGGCACGTATCATTGCCCTTATCTTGTGCAGTTAGACGCGAATTTTTC
GAAGAAGTACCTTCAAAGAATGGGGTCTCATCTTGTTTTGCAAGTACCACTGAGCAGGATAA
TAATAGAAATGATAATATACTATAGTAGAGATAACGTCGATGACTTCCCATACTGTAATTGC
TTTTAGTTGTGTATTTTTAGTGTGCAAGTTTCTGTAAATCGATTAATTTTTTTTTCTTTCCTCTT
TTTATTAACCTTAATTTTTATTTTAGATTCCTGACTTCAACTCAAGACGCACAGATATTATAAC
ATCTGCACAATAGGCATTTGCAAGAATTACTCGTGAGTAAGGAAAGAGTGAGGAACTATCGC
ATACCTGCATTTAAAGATGCCGATTTGGGCGCGAATCCTTTATTTTGGCTTCACCCTCATACT
ATTATCAGGGCCAGAAAAAGGAAGTGTTTCCCTCCTTCTTGAATTGATGTTACCCTCATAAAG
CACGTGGCCTCTTATCGAGAAAGAAATTACCGTCGCTCGTGATTTGTTTGCAAAAAGAACAA
AACTGAAAAAACCCAGACACGCTCGACTTCCTGTCTTCCTATTGATTGCAGCTTCCAATTTCG
TCACACAACAAGGTCCTAGCGACGGCTCACAGGTTTTGTAACAAGCAATCGAAGGTTCTGGA
ATGGCGGGAAAGGGTTTAGTACCACATGCTATGATGCCCACTGTGATCTCCAGAGCAAAGTT
CGTTCGATCGTACTGTTACTCTCTCTCTTTCAAACAGAATTGTCCGAATCGTGTGACAACAAC
AGCCTGTTCTCACACACTCTTTTCTTCTAACCAAGGGGGTGGTTTAGTTTAGTAGAACCTCGT
GAAACTTACATTTACATATATATAAACTTGCATAAATTGGTCAATGCAAGAAATACATATTTG
GTCTTTTCTAATTCGTAGTTTTTCAAGTTCTTAGATGCTTTCTTTTTCTCTTTTTTACAGATCAT
CAAGGAAGTAATTATCTACTTTTTACAACAAATATAAAACAATGAGCAGTAGCAAGAAATTG
GCCGGTCTTAGGGACAATTTCAGTTTGCTCGGCGAAAAGAATAAGATCTTGGTCGCCAATAG
AGGTGAAATTCCGATTAGAATTTTTAGATCTGCTCATGAGCTGTCTATGAGAACCATCGCCAT
ATACTCCCATGAGGACCGTCTTTCAATGCACAGGTTGAAGGCGGACGAAGCGTATGTTATCG

GGGAGGAGGGCCAGTATACACCTGTGGGTGCTTACTTGGCAATGGACGAGATCATCGAAATT
GCAAAGAAGCATAAGGTGGATTTCATCCATCCAGGTTATGGGTTCTTGTCTGAAAATTCGGA
ATTTGCCGACAAAGTAGTGAAGGCCGGTATCACTTGGATCGGCCCTCCAGCTGAAGTTATTG
ACTCTGTGGGTGACAAAGTCTCTGCCAGACACTTGGCAGCAAGAGCTAACGTTCCTACCGTT
CCCGGTACTCCAGGACCTATCGAAACTGTGCAAGAGGCACTTGACTTCGTTAATGAATACGG
CTACCCGGTGATCATTAAGGCCGCCTTTGGTGGTGGTGGTAGAGGTATGAGAGTCGTTAGAG
AAGGTGACGACGTGGCAGATGCCTTTCAACGTGCTACCTCCGAAGCCCGTACTGCCTTCGGT
AATGGTACCTGCTTTGTGGAAAGATTCTTGGACAAGCCAAAGCATATTGAAGTTCAATTGTT
GGCTGATAACCACGGAAACGTGGTTCATCTTTTCGAAAGAGACTGTTCTGTGCAAAGAAGAC
ACCAAAAAGTTGTCGAAGTCGCTCCAGCAAAGACTTTGCCCCGTGAAGTTCGTGACGCTATT
TTGACAGATGCTGTTAAATTAGCTAAGGTATGTGGTTACAGAAACGCAGGTACCGCCGAATT
CTTGGTTGACAACCAAAACAGACACTATTTCATTGAAATTAATCCAAGAATTCAAGTGGAGC
ATACCATCACTGAAGAAATCACCGGTATTGACATTGTTTCTGCCCAAATCCAGATTGCCGCA
GGTGCCACTTTGACTCAACTAGGTCTATTACAGGATAAAATCACCACCCGTGGGTTTTCCATC
CAATGTCGTATTACCACTGAAGATCCCTCTAAGAATTTCCAACCGGATACCGGTCGCCTGGA
GGTCTATCGTTCTGCCGGTGGTAATGGTGTGAGATTGGACGGTGGTAACGCTTATGCAGGTG
CTACTATCTCGCCTCACTACGACTCAATGCTGGTCAAATGTTCATGCTCTGGTTCTACTTATG
AAATCGTCCGTAGGAAGATGATTCGTGCCCTGATCGAATTCAGAATCAGAGGTGTTAAGACC
AACATTCCCTTCCTATTGACTCTTTTGACCAATCCAGTTTTTATTGAGGGTACATACTGGACG
ACTTTTATTGACGACACCCCACAACTGTTCCAAATGGTATCGTCACAAAACAGAGCGCAAAA
ACTGTTACACTATTTGGCAGACTTGGCAGTTAACGGTTCTTCTATTAAGGGTCAAATTGGCTT
GCCAAAACTAAAATCAAATCCAAGTGTCCCCCATTTGCACGATGCTCAGGGCAATGTCATCA
ACGTTACAAAGTCTGCACCACCATCCGGATGGAGACAAGTGCTACTGGAAAAGGGACCATCT
GAATTTGCCAAGCAAGTCAGACAGTTCAATGGTACTCTACTGATGGACACCACCTGGAGAGA
CGCTCATCAATCTCTACTTGCAACAAGAGTCAGAACCCACGATTTGGCTACAATCGCTCCAA
CAACCGCACATGCCCTTGCAGGTGCTTTCGCTTTAGAATGTTGGGGTGGTGCTACATTCGACG
TTGCAATGAGATTCTTGCATGAGGATCCATGGGAACGTCTGAGAAAATTAAGATCTCTGGTG
CCTAATATTCCATTCCAAATGTTATTACGTGGTGCCAACGGTGTGGCTTACTCTTCATTACCT
GACAATGCTATTGACCATTTTGTCAAGCAAGCCAAGGATAATGGTGTTGATATATTTAGAGTT
TTTGATGCCTTGAATGATTTAGAACAATTAAAAGTTGGTGTGAATGCTGTCAAGAAGGCCGG
TGGTGTTGTCGAAGCTACTGTTTGTTACTCTGGTGACATGCTTCAGCCAGGTAAGAAATACAA
CTTAGACTACTACCTAGAAGTTGTTGAAAAAATAGTTCAAATGGGTACACATATCTTGGGTA
TTAAGGATATGGCAGGTACTATGAAACCGGCCGCTGCCAAATTATTAATTGGCTCCCTAAGA
ACCAGATATCCGGATTTACCAATTCATGTTCACAGTCATGACTCCGCAGGTACTGCTGTTGCG
TCTATGACTGCATGTGCCCTAGCAGGTGCTGATGTTGTCGATGTAGCTATCAATTCAATGTCG
GGCTTAACTTCCCAACCATCAATTAATGCACTGTTGGCTTCATTAGAAGGTAACATTGATACT
GGGATTAACGTTGAGCATGTTCGTGAATTAGATGCATACTGGGCCGAAATGAGACTGTTGTA
TTCTTGTTTCGAGGCCGACTTGAAGGGACCAGATCCAGAAGTTTACCAACATGAAATCCCAG
GTGGTCAATTGACTAACTTGTTATTCCAAGCTCAACAACTGGGTCTTGGTGAACAATGGGCTG
AAACTAAAAGAGCTTACAGAGAAGCCAATTACCTACTGGGAGATATTGTTAAAGTTACCCCA
ACTTCTAAGGTTGTCGGTGATTTAGCTCAATTCATGGTTTCTAACAAACTGACTTCCGACGAT
ATTAGACGTTTAGCTAATTCTTTGGACTTTCCTGACTCTGTTATGGACTTTTTTGAAGGTTTAA
TTGGTCAACCATACGGTGGGTTCCCAGAACCATTAAGATCTGATGTATTGAGAAACAAGAGA
AGAAAGTTGACGTGCCGTCCAGGTTTAGAATTAGAACCATTTGATCTCGAAAAAATTAGAGA
AGACTTGCAGAACAGATTCGGTGATATTGATGAATGCGATGTTGCTTCTTACAATATGTATCC

AAGGGTCTATGAAGATTTCCAAAAGATCAGAGAAACATACGGTGATTTATCAGTTCTACCAA
CCAAAAATTTCCTAGCACCAGCAGAACCTGATGAAGAAATCGAAGTCACCATCGAACAAGG
TAAGACTTTGATTATCAAATTGCAAGCTGTTGGTGACTTAAATAAGAAAACTGGGCAAAGAG
AAGTGTATTTTGAATTGAACGGTGAATTAAGAAAGATCAGAGTTGCAGACAAGTCACAAAAC
ATACAATCTGTTGCTAAACCAAAGGCTGATGTCCACGATACTCACCAAATCGGTGCACCAAT
GGCTGGTGTTATCATAGAAGTTAAAGTACATAAAGGGTCTTTGGTGAAAAAGGGCGAATCGA
TTGCTGTTTTGAGTGCCATGAAAATGGAAATGGTTGTCTCTTCACCAGCAGATGGTCAAGTTA
AAGACGTTTTCATTAAGGATGGTGAAAGTGTTGACGCATCAGATTTGTTGGTTGTCCTAGAA
GAAGAAACCCTACCCCCATCCCAAAAAAAGTAAATTGAATTGAATTGAAATCGATAGATCAA
TTTTTTTCTTTTCTCTTTCCCCATCCTTTACGCTAAAATAATAGTTTATTTTATTTTTTGAATAT
TTTTTATTTATATACGTATATATAGACTATTATTTATCTTTTAATGATTATTAAGATTTTTATTA
AAAAAAAATTCGCTCCTCTTTTAATGCCTTTATGCAGTTTTTTTTTCCCATTCGATATTTCTAT
GT

2. PCK (Promoter ENO2 – PCK – Terminator ENO2)

GTGTCGACGCTGCGGGTATAGAAAGGGTTCTTTACTCTATAGTACCTCCTCGCTCAGCATCTG
CTTCTTCCCAAAGATGAACGCGGCGTTATGTCACTAACGACGTGCACCAACTTGCGGAAAGT
GGAATCCCGTTCCAAAACTGGCATCCACTAATTGATACATCTACACACCGCACGCCTTTTTTC
TGAAGCCCACTTTCGTGGACTTTGCCATATGCAAAATTCATGAAGTGTGATACCAAGTCAGC
ATACACCTCACTAGGGTAGTTTCTTTGGTTGTATTGATCATTTGGTTCATCGTGGTTCATTAAT
TTTTTTTCTCCATTGCTTTCTGGCTTTGATCTTACTATCATTTGGATTTTTGTCGAAGGTTGTAG
AATTGTATGTGACAAGTGGCACCAAGCATATATAAAAAAAAAAAGCATTATCTTCCTACCAG
AGTTGATTGTTAAAAACGTATTTATAGCAAACGCAATTGTAATTAATTCTTATTTTGTATCTTT
TCTTCCCTTGTCTCAATCTTTTATTTTTATTTTATTTTTCTTTTCTTAGTTTCTTTCATAACACCA
AGCAACTAATACTATAACATACAATAATAATGACCGATTTGAATCAATTGACCCAAGAATTG
GGTGCCTTGGGTATTCATGATGTTCAAGAAGTTGTTTACAACCCATCCTACGAATTGTTGTTC
GCTGAAGAAACAAAACCAGGTTTGGAAGGTTACGAAAAGGGTACTGTTACTAATCAAGGTG
CTGTTGCTGTTAACACTGGTATTTTTACTGGTAGATCCCCAAAGGATAAGTACATCGTTTTGG
ATGATAAGACCAAGGATACTGTTTGGTGGACTTCTGAAAAGGTTAAGAACGATAACAAGCCA
ATGTCTCAAGATACCTGGAATTCTTTGAAAGGTTTGGTTGCCGATCAATTGTCCGGTAAGAGA
TTATTTGTTGTTGATGCTTTCTGCGGTGCTAACAAAGATACCAGATTGGCTGTTAGAGTTGTT
ACTGAAGTTGCATGGCAAGCTCATTTTGTTACCAACATGTTCATTAGACCATCCGCCGAAGA
ATTGAAGGGTTTTAAACCAGATTTCGTCGTTATGAACGGTGCTAAGTGTACTAATCCAAACTG
GAAAGAACAAGGTTTGAATTCCGAAAACTTCGTCGCTTTCAACATTACCGAAGGTGTTCAAT
TGATAGGTGGTACTTGGTATGGTGGTGAAATGAAGAAAGGTATGTTCTCCATGATGAACTAC
TTTTTGCCATTGAGAGGTATTGCCTCTATGCATTGCTCTGCTAACGTTGGTAAAGATGGTGAT
ACTGCTATTTTCTTCGGTTTGTCTGGTACTGGTAAGACTACTTTGTCTACTGATCCTAAGAGAC
AATTGATTGGTGATGATGAACACGGTTGGGACGATGAAGGTGTTTTTAACTTTGAAGGTGGT
TGTTACGCCAAGACCATTAACTTGTCTGCTGAAAATGAACCAGATATCTACGGTGCCATTAA
GAGAGATGCTTTGTTGGAAAACGTTGTCGTCTTGGATAATGGTGATGTTGATTATGCTGATGG
TTCCAAGACTGAAAACACCAGAGTTTCTTATCCAATCTACCATATCCAAAACATCGTCAAGC
CAGTTTCTAAAGCTGGTCCAGCTACAAAGGTTATCTTTTTATCTGCTGATGCCTTCGGTGTTTT
GCCACCAGTTTCAAAATTGACTCCAGAACAAACTAAGTACTACTTCTTGTCTGGTTTCACTGC
TAAATTGGCTGGTACTGAAAGAGGTATTACTGAACCTACTCCAACTTTCTCTGCTTGTTTTGG

TGCTGCTTTTTTGTCATTGCATCCAACTCAATACGCTGAAGTTTTGGTCAAGAGAATGCAAGA
ATCTGGTGCTGAAGCTTACTTGGTTAATACTGGTTGGAATGGTACAGGTAAAAGAATCTCCA
TTAAGGACACCAGAGGTATCATTGATGCTATCTTGGATGGTTCTATTGACAAGGCTGAAATG
GGTTCCTTGCCAATTTTCGATTTCTCTATTCCAAAAGCCTTGCCAGGTGTTAACCCAGCTATTT
TAGATCCAAGAGATACCTATGCTGATAAGGCTCAATGGGAAGAAAAAGCTCAAGATTTGGC
AGGTAGATTCGTCAAGAATTTCGAAAAGTATACCGGTACTGCTGAAGGTCAAGCTTTGGTTG
CTGCTGGTCCAAAAGCTTAAAGTGCTTTTAACTAAGAATTATTAGTCTTTTCTGCTTATTTTTT
CATCATAGTTTAGAACACTTTATATTAACGAATAGTTTATGAATCTATTTAGGTTTAAAAATT
GATACAGTTTTATAAGTTACTTTTTCAAAGACTCGTGCTGTCTATTGCATAATGCACTGGAAG
GGGAAAAAAAAGGTGCACACGCGTGGCTTTTTCTTGAATTTGCAGTTTGAAAAAT

3. MDH (Promoter TPI – MDH – Terminator ADH1)

TATATCTAGGAACCCATCAGGTTGGTGGAAGATTACCCGTTCTAAGACTTTTCAGCTTCCTCT
ATTGATGTTACACCTGGACACCCCTTTTCTGGCATCCAGTTTTTAATCTTCAGTGGCATGTGA
GATTCTCCGAAATTAATTAAAGCAATCACACAATTCTCTCGGATACCACCTCGGTTGAAACTG
ACAGGTGGTTTGTTACGCATGCTAATGCAAAGGAGCCTATATACCTTTGGCTCGGCTGCTGTA
ACAGGGAATATAAAGGGCAGCATAATTTAGGAGTTTAGTGAACTTGCAACATTTACTATTTT
CCCTTCTTACGTAAATATTTTTCTTTTTAATTCTAAATCAATCTTTTTCAATTTTTTGTTTGTAT
TCTTTTCTTGCTTAAATCTATAACTACAAAAAACACATACATAAACTAAAAATGGTCAAAGTC
GCAATTCTTGGCGCTTCTGGTGGCGTGGGACAACCGCTATCATTACTGCTAAAATTAAGCCCT
TACGTTTCCGAGCTGGCGTTGTACGATATCCGAGCTGCGGAAGGCATTGGTAAGGATTTATCT
CACATCAACACCAACTCAAGTTGTGTCGGTTATGATAAGGATAGTATTGAGAACACCTTGTC
AAATGCTCAGGTGGTGCTAATACCGGCTGGTGTTCCCAGAAAGCCCGGTTTAACTAGAGATG
ATTTGTTCAAGATGAACGCCGGTATTGTCAAAAGCCTGGTAACCGCTGTTGGAAAGTTCGCA
CCAAATGCGAGGATTTTAGTCATTTCAAACCCTGTAAACAGTTTGGTCCCTATTGCTGTGGAA
ACTTTGAAGAAAATGGGTAAGTTCAAACCTGGAAACGTTATGGGTGTGACGAACCTTGACCT
GGTACGTGCAGAAACCTTTTTGGTAGATTATTTGATGCTAAAAAACCCCAAAATTGGACAAG
AACAAGACAAAACTACAATGCACAGAAAGGTCACTGTTATTGGGGGTCATTCAGGGGAAAC
CATTATCCCAATAATCACCGACAAATCGCTGGTATTTCAACTTGATAAGCAGTACGAGCACTT
CATTCATAGGGTCCAGTTCGGAGGTGATGAAATTGTCAAAGCTAAACAGGGCGCCGGTTCCG
CCACGTTGTCCATGGCGTTCGCGGGGGCCAAGTTTGCTGAAGAAGTTTTGAGGAGCTTCCAT
AATGAGAAACCAGAAACGGAGTCACTTTCCGCATTCGTTTATTTACCAGGCTTAAAAAACGG
TAAGAAAGCGCAGCAATTAGTTGGCGACAACTCTATTGAGTATTTTTCCTTGCCAATTGTTTT
GAGAAATGGTAGCGTAGTATCCATCGATACCAGTGTTCTGGAAAAACTGTCTCCGAGAGAGG
AACAACTCGTTAATACTGCGGTCAAAGAGCTACGCAAGAATATTGAAAAAGGCAAGAGTTTC
ATCCTAGACTCTTCCTAAGCGAATTTCTTATGATTTATGATTTTTATTATTAAATAAGTTATAA
AAAAAATAAGTGTATACAAATTTTAAAGTGACTCTTAGGTTTTAAAACGAAAATTCTTATTCT
TGAGTAACTCTTTCCTGTAGGTCAGGTTGCTTTCTCAGGTATAGCATGAGGTCGCTCTTATTG
ACCACACCTCTACCGGCATGCCGAGCAAATGCCTGCAAATCGCTCCCCATTTC

4. FUMC (Promoter TDH3 – FUMC – Terminator CYC1)

TTAGTCAAAAAATTAGCCTTTTAATTCTGCTGTAACCCGTACATGCCCAAAATAGGGGGCGG
GTTACACAGAATATATAACATCGTAGGTGTCTGGGTGAACAGTTTATTCCTGGCATCCACTAA
ATATAATGGAGCCCGCTTTTTAAGCTGGCATCCAGAAAAAAAAAGAATCCCAGCACCAAAAT
ATTGTTTTCTTCACCAACCATCAGTTCATAGGTCCATTCTCTTAGCGCAACTACAGAGAACAG

GGGCACAAACAGGCAAAAAACGGGCACAACCTCAATGGAGTGATGCAACCTGCCTGGAGTA
AATGATGACACAAGGCAATTGACCCACGCATGTATCTATCTCATTTTCTTACACCTTCTATTA
CCTTCTGCTCTCTCTGATTTGGAAAAAGCTGAAAAAAAAGGTTGAAACCAGTTCCCTGAAAT
TATTCCCCTACTTGACTAATAAGTATATAAAGACGGTAGGTATTGATTGTAATTCTGTAAATC
TATTTCTTAAACTTCTTAAATTCTACTTTTATAGTTAGTCTTTTTTTTAGTTTTAAAACACCAA
GAACTTAGTTTCGAATAAACACACATAAACAAACAAAATGAACACCGTTAGATCCGAAAAG
GATTCCATGGGTGCTATTGATGTTCCAGCTGATAAGTTGTGGGGTGCTCAAACTCAAAGATCC
TTGGAACATTTCAGAATCTCCACTGAAAAGATGCCAACCTCTTTGATTCATGCTTTGGCTTTG
ACAAAAAGAGCTGCTGCTAAGGTTAATGAAGATTTGGGTTTGTTGTCCGAAGAAAAGGCTTC
TGCTATTAGACAAGCTGCTGATGAAGTTTTGGCTGGTCAACATGATGATGAATTCCCATTGGC
TATTTGGCAAACTGGTTCTGGTACTCAATCTAACATGAACATGAATGAAGTCTTGGCCAACA
GAGCCTCTGAATTATTGGGTGGTGTTAGAGGTATGGAAAGAAAGGTTCATCCAAACGATGAT
GTCAACAAGTCCCAATCTTCTAACGATGTTTTTCCAACCGCTATGCATGTTGCTGCTTTGTTG
GCATTGAGAAAGCAATTGATCCCTCAATTGAAAACCTTGACCCAAACCTTGAACGAAAAGTC
AAGAGCTTTTGCCGATATCGTTAAGATTGGTAGAACTCACTTGCAAGATGCTACACCATTGA
CTTTAGGTCAAGAAATTTCAGGTTGGGTTGCCATGTTGGAACATAACTTGAAGCACATCGAA
TACTCATTGCCACATGTTGCAGAATTGGCTTTGGGTGGTACTGCTGTTGGTACTGGTTTAAAT
ACTCATCCAGAATACGCTAGAAGAGTTGCAGATGAATTGGCTGTTATTACTTGTGCTCCATTT
GTTACTGCTCCAAACAAGTTTGAAGCTTTGGCTACTTGTGATGCTTTGGTTCAAGCTCATGGT
GCTTTGAAAGGTTTGGCTGCTTCTTTGATGAAGATTGCCAATGATGTTAGATGGTTGGCTTCT
GGTCCAAGATGTGGTATTGGTGAAATTTCCATTCCAGAAAACGAACCAGGTTCCTCTATTATG
CCAGGTAAAGTTAATCCAACCCAATGCGAAGCTTTGACCATGTTGTGTTGCCAAGTTATGGG
TAATGATGTTGCCATCAATATGGGTGGTGCTTCTGGTAATTTTGAATTGAACGTTTTCAGACC
AATGGTTATCCACAACTTCTTGCAATCCGTTAGATTATTGGCTGACGGTATGGAATCTTTCAA
CAAACATTGTGCCGTTGGTATCGAACCTAACAGAGAAAGAATCAATCAATTATTGAACGAAT
CCTTGATGTTGGTCACTGCTTTGAACACTCACATTGGTTATGATAAGGCTGCTGAAATTGCTA
AGAAGGCTCACAAAGAAGGTTTGACTTTGAAAGCTGCTGCATTAGCTTTGGGTTATTTGTCTG
AAGCTGAATTCGATTCTTGGGTCAGACCAGAACAAATGGTTGGTTCTATGAAGGCTGGTAGA
TGAATCCGCTCTAACCGAAAAGGAAGGAGTTAGACAACCTGAAGTCTAGGTCCCTATTTATT
TTTTTATAGTTATGTTAGTATTAAGAACGTTATTTATATTTCAAATTTTTCTTTTTTTTCTGTAC
AGACGCGTGTACGCATGTAACATTATACTGAAAACCTTGCTTGAGAAGGTTTTGGGACGCTC
GAAG

5. FRD (Promoter TEF1 – FRD – Terminator TDH1 – URA3 – X2 sequence)

ATAGCTTCAAAATGTTTCTACTCCTTTTTTACTCTTCCAGATTTTCTCGGACTCCGCGCATCGC
CGTACCACTTCAAAACACCCAAGCACAGCATACTAAATTTCCCCTCTTTCTTCCTCTAGGGTG
TCGTTAATTACCCGTACTAAAGGTTTGGAAAAGAAAAAAGAGACCGCCTCGTTTCTTTTTCTT
CGTCGAAAAAGGCAATAAAAATTTTTATCACGTTTCTTTTTCTTGAAAATTTTTTTTTTTGATT
TTTTTCTCTTTCGATGACCTCCCATTGATATTTAAGTTAATAAACGGTCTTCAATTTCTCAAGT
TTCAGTTTCATTTTTCTTGTTCTATTACAACTTTTTTTACTTCTTGCTCATTAGAAAGAAAGCA
TAGCAATCTAATCTAAGTTTTAATTACAAAATGTCTCTCTCTCCCGTTGTTGTTATTGGAACCG
GTTTGGCCGGGCTGGCTGCTGCCAATGAATTGGTTAACAAGTATAACATCCCTGTAACCATCC
TCGAAAAGGCTTCCTCGATCGGTGGGAACTCTATCAAGGCCTCCAGTGGTATTAACGGTGCT

TGCACCGAGACTCAACGTCACTTCCACATCGAGGACTCCCCACGCTTATTTGAAGATGACAC
CATCAAGTCTGCTAAAGGTAAAGGTGTCCAAGAATTAATGGCTAAGTTGGCCAATGATTCTC
CCCTGGCTATTGAATGGTTGAAAAACGAATTTGATTTGAAATTGGACCTATTGGCTCAATTGG
GTGGCCACTCTGTGGCAAGAACTCACAGATCGTCTGGGAAGTTGCCTCCAGGTTTCGAAATT
GTTTCTGCCTTATCTAACAATTTGAAGAAATTAGCTGAGACTAAACCAGAGTTAGTTAAGATT
AACTTAGACAGTAAAGTCGTAGACATCCATGAAAAGGATGGCTCCATTTCTGCTGTAGTGTA
CGAGGACAAGAATGGCGAAAAGCACATGGTGAGTGCTAACGATGTCGTTTTTTGTTCTGGAG
GGTTTGGCTTTTCTAAGGAAATGCTTAAAGAATACGCACCCGAACTGGTGAACTTGCCAACA
ACAAACGGGCAACAAACAACTGGTGATGGTCAAAGGCTTCTGCAGAAGTTAGGCGCTGATCT
GATTGACATGGACCAAATTCAAGTTCATCCAACTGGGTTCATTGATCCAAATGACCGTAGCT
CAAGCTGGAAATTCTTGGCTGCCGAATCCTTAAGAGGTCTTGGTGGTATCCTATTAAACCCTA
TTACCGGTAGAAGATTTGTCAACGAATTGACCACAAGAGATGTAGTCACTGCAGCTATTCAA
AAGGTTTGTCCTCAAGAGGATAACAGAGCACTATTGGTTATGGGCGAAAAAATGTACACAGA
TTTGAAGAATAATTTAGATTTTTACATGTTCAAGAAACTTGTACAGAAATTGACATTATCTCA
AGTTGTTTCTGAATATAATTTACCAATCACTGTCGCCCAATTATGCGAGGAATTGCAAACATA
CTCTTCCTTCACTACCAAGGCTGATCCGTTGGGACGTACCGTTATTCTCAACGAATTTGGCTC
TGACGTTACTCCAGAAACTGTGGTTTTTATTGGTGAAGTAACACCGGTTGTCCATTTCACCAT
GGGTGGTGCTAGAATCAATGTCAAGGCTCAAGTCATTGGCAAGAACGACGAAAGGCTACTA
AAAGGCCTGTACGCGGCCGGTGAAGTTTCTGGCGGTGTTCATGGCGCCAATAGGTTGGGTGG
TTCAAGTTTGTTAGAATGCGTTGTCTTTGGGAGAACCGCAGCTGAATCTATTGCCAATGACCG
CAAGTAAATAAAGCAATCTTGATGAGGATAATGATTTTTTTTTGAATATACATAAATACTACC
GTTTTTCTGCTAGATTTTGTGAAGACGTAAATAAGTACATATTACTTTTTAAGCCAAGACAAG
ATTAAGCATTAACTTTACCCTTTTCTCTTCTAAGTTTCAATACTAGTTATCACTGTTTAAAAGT
TATGGCGAGAACGTCGGCGGTTAAAATATATTACCCTGAACGATCGCGTCAGCTGAAGCTTC
GTACGCTGCAGGTCGACAACCCTTAATATAACTTCGTATAATGTATGCTATACGAAGTTATTA
GGTCTAGAGATCCCAATACAACAGATCACGTGATCTTTTGTAAGATGAAGTTGAAGTGAGTG
TTGCACCGTGCCAATGCAGGTGGCTATTAGATTAAATATGTGATTTGTTCTATTAAGTTTCCT
GTATAATTAATGGGGAGCGCTGATTCTCTTTTGGTACGCTTCCCATCCAGCATTTCTGTATCTT
TCACCTTCAACCTTAGGATCTCTACCCTTGGCGAAAAGTCCTCTGCCAACAATGATGATATCT
GATCCACCACTTACAACTTCGTCGACGGTTCTGTACTGCTGACCCAATGCATCGCCTTTGTCG
TCTAAACCTACACCTGGGGTCATGATTAGCCAATCAAACCCTTCTTCTCTTCCTCCCATATCG
TTCTGAGCAATGAACCCAATAACGAAATCTTTATCACTCTTTGCAATATCAACGGTACCCTTA
GTATATTCACCGTGTGCTAGAGAACCCTTGGAAGACAATTCAGCAAGCATCAATAATCCCCT
TGGTTCTTTGGTGACCTCTTGCGCACCTTGTTTCAAGCCAGCAACAATACCAGCACCAGTAAC
CCCGTGGGCGTTGGTGATATCAGACCATTCTGCGATACGGTAAACGCCCGATGTATATTGTA
ATTTGACTGTGTTACCGATATCGGCGAATTTTCTGTCCTCAAATATCAAGAACTTGTATTTCTC
TGCCAATGCTTTCAATGGAACGACAGTACCCTCATAACTGAAATCATCCAAGATATCAACGT
GTGTTTTCAAAAGGCAAATGTATGGACCCAACGTTTCAACAAGTTTCAATAGCTCATCAGTC
GAACGAACGTCAAGAGAAGCACACAAATTGGTCTTCTTTTCATCCATTAAACGTAAAAGTTT
CGATGCAACCGGACTTGCATGAGTCTCAGCTCTACTGGTATATGATTTTGTGGACATGGTGCA
ACTAATTGACGGGAGTGTATTGACGCTGGCGTACTGGCTTTCACAAAATGGCCCAATCACAA
CCACATCTTAGATAGTTGAAATGACTTTAGATAACATCAATTGAGATGAGCTTAATCATGTCA
AAGCTAAAAGTGTCACCATGAACGACAATTCTTAAGCAAATCACGTGATATAGATCCACGAA
TAACCACCATTTGATGCTCGAGGCAAGTAATGTGTGTAAAAAAATGCGTTACCACCATCCAA
TGCAGACCGATCTTCTACCCAGAATCACATATATTTATGTACCGAGTACCTTTTTTCTATCTTC

CAATTGCTTCTCCCATATGATTGTCTCCGTAAGCTCGAAATTTCTAAGTTGGATTTTAATCTTC
ACGCAGGATGACAGTTCGATGAGCTTCTGAGGAGTGTTTAGAACATAATCAGTTTATCCATG
GTCTATCTCTTCTTGTCGCTTTTTCTCCTCGATAGAACCTAAATAAAACGAGCTCTCGAGAAC
CCTTAATATAACTTCGTATAATGTATGCTATACGAAGTTATTAGGTGATATCAGATCCACTAG
TGGCCTATGCACCCAATTCGCCCTATAGTGAGTCGTATTACGCGCGCTCACTGGCCGTCGTTT
TACAACGTCGTGACTGGGAAAACCCTGGCGTTACCCCTGCAGGACTAGTGCTGAGGCATTAA
TCCTGCATAATCGGCCTCACAGAGGGATCCCGTTACCCATCTATGCTGAAGATTTATCATACT
ATTCCTCCGCTCGTTTCTTTTTTCAGTGAGGTGTGTCGTGAAAGAAAACCCACAATTAAACTT
TCAACAACCGGGCGACTAGGAAGAGAGTAGTGGGCGCGGATGACGAAGGCTAAGGTCACTT
CTTCGTTTCCTTTATTGGGGTTTCCGTGTAGCCTTCCCCTGAATAGTGTGGGACGTTTTATGAG
AAGCCGTAAGAAATAGGCAAATTGAGTTATGACAAGTAGACATGATGCCGCAGCCTTGCCTG
ACTTTACGTCTCCTTCATGAATAAGTTTTTCTATCGAGTTCTTTTCCTTTTTTCGCCTTAATTAG
CTCAATTAAGCCTGTCCTCACTACTTTTCTTTTTCTTATCGGCTTTGTGCCACACCTAACCTTC
GAATGCTGTTTTATTCCGTTCTTACATGGGATGGTAATGCCTTGGCGAG

6. *mae1* (Promoter TEF2 – *mae1* – Terminator ENO1)

GGGGCCGTATACTTACATATAGTAGATGTCAAGCGTAGGCGCTTCCCCTGCCGGCTGTGAGG
GCGCCATAACCAAGGTATCTATAGACCGCCAATCAGCAAACTACCTCCGTACATTCATGTTG
CACCCACACATTTATACACCCAGACCGCGACAAATTACCCATAAGGTTGTTTGTGACGGCGT
CGTACAAGAGAACGTGGGAACTTTTTAGGCTCACCAAAAAAGAAAGAAAAAATACGAGTTG
CTGACAGAAGCCTCAAGAAAAAAAAAATTCTTCTTCGACTATGCTGGAGGCAGAGATGATCG
AGCCGGTAGTTAACTATATATAGCTAAATTGGTTCCATCACCTTCTTTTCTGGTGTCGCTCCTT
CTAGTGCTATTTCTGGCTTTTCCTATTTTTTTTTTTCCATTTTTCTTTCTCTCTTTCTAATATATA
AATTCTCTTGCATTTTCTATTTTTCTCTCTATCTATTCTACTTGTTTATTCCCTTCAAGGTTTTTT
TTTAAGGAGTACTTGTTTTTAGAATATACGGTCAACGAACTATAATTAACTAAACATGGGTG
AACTCAAGGAAATCTTGAAACAGAGGTATCATGAGTTGCTTGACTGGAATGTCAAAGCCCCT
CATGTCCCTCTCAGTCAACGACTGAAGCATTTTACATGGTCTTGGTTTGCATGTACTATGGCA
ACTGGTGGTGTTGGTTTGATTATTGGTTCTTTCCCCTTTCGATTTTATGGTCTTAATACAATTG
GCAAAATTGTTTATATTCTTCAAATCTTTTTGTTTTCTCTCTTTGGATCATGCATGCTTTTTCGC
TTTATTAAATATCCTTCAACTATCAAGGATTCCTGGAACCATCATTTGGAAAAGCTTTTCATT
GCTACTTGTCTTCTTTCAATATCCACGTTCATCGACATGCTTGCCATATACGCCTATCCTGATA
CCGGCGAGTGGATGGTGTGGGTCATTCGAATCCTTTATTACATTTACGTTGCAGTATCCTTTA
TATACTGCGTAATGGCTTTTTTTACAATTTTCAACAACCATGTATATACCATTGAAACCGCAT
CTCCTGCTTGGATTCTTCCTATTTTCCCTCCTATGATTTGTGGTGTCATTGCTGGCGCCGTCAA
TTCTACACAACCCGCTCATCAATTAAAAAATATGGTTATCTTTGGTATCCTCTTTCAAGGACT
TGGTTTTTGGGTTTATCTTTTACTGTTTGCCGTCAATGTCTTACGGTTTTTTACTGTAGGCCTG
GCAAAACCCCAAGATCGACCTGGTATGTTTATGTTTGTCGGTCCACCAGCTTTCTCAGGTTTG
GCCTTAATTAATATTGCGCGTGGTGCTATGGGCAGTCGCCCTTATATTTTTGTTGGCGCCAAC
TCATCCGAGTATCTTGGTTTTGTTTCTACCTTTATGGCTATTTTTATTTGGGGTCTTGCTGCTTG
GTGTTACTGTCTCGCCATGGTTAGCTTTTTAGCGGGCTTTTTCACTCGAGCCCCTCTCAAGTTT
GCTTGTGGATGGTTTGCATTCATTTTCCCCAACGTGGGTTTTGTTAATTGTACCATTGAGATA
GGTAAAATGATAGATTCCAAAGCTTTCCAAATGTTTGGACATATCATTGGGGTCATTCTTTGT
ATTCAGTGGATCCTCCTAATGTATTTAATGGTCCGTGCGTTTCTCGTCAATGATCTTTGCTATC
CTGGCAAAGACGAAGATGCCCATCCTCCACCAAAACCAAATACAGGTGTCCTTAACCCTACC
TTCCCACCTGAAAAAGCACCTGCATCTTTGGAAAAAGTCGATACACATGTCACATCTACTGG

TGGTGAATCGGATCCTCCTAGTAGTGAACATGAAAGCGTTTAAAGCTTTTGATTAAGCCTTCT
AGTCCAAAAAACACGTTTTTTTGTCATTTATTTCATTTTCTTAGAATAGTTTAGTTTATTCATT
TTATAGTCACGAATGTTTTATGATTCTATATAGGGTTGCAAACAAGCATTTTTCATTTTATGTT
AAAACAATTTCAGGTTTACCTTTTATTCTGCTTGTGGTGACGCGTGTATCCGCCCGCTCTTTTG
GTCACCCATGTAT

7. Pyc-Ty (**Ty1Cons2-5'** – Promoter pGK1 – PYC2 – Terminator PGK1 – LEU Marker with degradation signal – **Ty1Cons2-3'**)

**TGTTGGAATAAAAATCAACTATCATCTACTAACTAGTATTTACGTTACTAGTATATTATC
ATATACGGTGTTAGAAGATGACGCAAATGATGAGAAATAGTCATCTAAATTAGTGGAAG
CTGAAACGCAAGGATTGATAATGTAATAGGATCATGAATATTAACATA**GCTAGCATAAGT
CCTCAGCGAGCTCGCATGGAATGCGTCAGGCATGAACGCATCACAGACAAAATCTTCTTGAC
AAACGTCACAATTGATCCCTCCCCATCCGTTATCACAATGACAGGTGTCATTTTGTGCTCTTA
TGGGACGATCCTTATTACCGCTTTCATCCGGTGATAGACCGCCACAGAGGGGCAGAGAGCAA
TCATCACCTGCAAACCCTTCTATACACTCACATCTACCAGTGTACGAATTGCATTCAGAAAAC
TGTTTGCATTCAAAAATAGGTAGCATACAATTAAAACATGGCGGGCACGTATCATTGCCCTT
ATCTTGTGCAGTTAGACGCGAATTTTTCGAAGAAGTACCTTCAAAGAATGGGGTCTCATCTTG
TTTTGCAAGTACCACTGAGCAGGATAATAATAGAAATGATAATATACTATAGTAGAGATAAC
GTCGATGACTTCCCATACTGTAATTGCTTTTAGTTGTGTATTTTTAGTGTGCAAGTTTCTGTAA
ATCGATTAATTTTTTTTTCTTTCCTCTTTTTATTAACCTTAATTTTTATTTTAGATTCCTGACTTC
AACTCAAGACGCACAGATATTATAACATCTGCACAATAGGCATTTGCAAGAATTACTCGTGA
GTAAGGAAAGAGTGAGGAACTATCGCATACCTGCATTTAAAGATGCCGATTTGGGCGCGAAT
CCTTTATTTTGGCTTCACCCTCATACTATTATCAGGGCCAGAAAAAGGAAGTGTTTCCCTCCT
TCTTGAATTGATGTTACCCTCATAAAGCACGTGGCCTCTTATCGAGAAAGAAATTACCGTCGC
TCGTGATTTGTTTGCAAAAAGAACAAAACTGAAAAAACCCAGACACGCTCGACTTCCTGTCT
TCCTATTGATTGCAGCTTCCAATTTCGTCACACAACAAGGTCCTAGCGACGGCTCACAGGTTT
TGTAACAAGCAATCGAAGGTTCTGGAATGGCGGGAAAGGGTTTAGTACCACATGCTATGATG
CCCACTGTGATCTCCAGAGCAAAGTTCGTTCGATCGTACTGTTACTCTCTCTCTTTCAAACAG
AATTGTCCGAATCGTGTGACAACAACAGCCTGTTCTCACACACTCTTTTCTTCTAACCAAGGG
GGTGGTTTAGTTTAGTAGAACCTCGTGAAACTTACATTTACATATATATAAACTTGCATAAAT
TGGTCAATGCAAGAAATACATATTTGGTCTTTTCTAATTCGTAGTTTTTCAAGTTCTTAGATGC
TTTCTTTTTCTCTTTTTTACAGATCATCAAGGAAGTAATTATCTACTTTTTACAACAAATATAA
AACAATGAGCAGTAGCAAGAAATTGGCCGGTCTTAGGGACAATTTCAGTTTGCTCGGCGAAA
AGAATAAGATCTTGGTCGCCAATAGAGGTGAAATTCCGATTAGAATTTTTAGATCTGCTCAT
GAGCTGTCTATGAGAACCATCGCCATATACTCCCATGAGGACCGTCTTTCAATGCACAGGTT
GAAGGCGGACGAAGCGTATGTTATCGGGGAGGAGGGCCAGTATACACCTGTGGGTGCTTACT
TGGCAATGGACGAGATCATCGAAATTGCAAAGAAGCATAAGGTGGATTTCATCCATCCAGGT
TATGGGTTCTTGTCTGAAAATTCGGAATTTGCCGACAAAGTAGTGAAGGCCGGTATCACTTG
GATCGGCCCTCCAGCTGAAGTTATTGACTCTGTGGGTGACAAAGTCTCTGCCAGACACTTGG
CAGCAAGAGCTAACGTTCCTACCGTTCCCGGTACTCCAGGACCTATCGAAACTGTGCAAGAG
GCACTTGACTTCGTTAATGAATACGGCTACCCGGTGATCATTAAGGCCGCCTTTGGTGGTGGT
GGTAGAGGTATGAGAGTCGTTAGAGAAGGTGACGACGTGGCAGATGCCTTTCAACGTGCTAC
CTCCGAAGCCCGTACTGCCTTCGGTAATGGTACCTGCTTTGTGGAAAGATTCTTGGACAAGCC
AAAGCATATTGAAGTTCAATTGTTGGCTGATAACCACGGAAACGTGGTTCATCTTTTCGAAA
GAGACTGTTCTGTGCAAAGAAGACACCAAAAAGTTGTCGAAGTCGCTCCAGCAAGACTTTG

CCCCGTGAAGTTCGTGACGCTATTTTGACAGATGCTGTTAAATTAGCTAAGGTATGTGGTTAC
AGAAACGCAGGTACCGCCGAATTCTTGGTTGACAACCAAAACAGACACTATTTCATTGAAAT
TAATCCAAGAATTCAAGTGGAGCATACCATCACTGAAGAAATCACCGGTATTGACATTGTTT
CTGCCCAAATCCAGATTGCCGCAGGTGCCACTTTGACTCAACTAGGTCTATTACAGGATAAA
ATCACCACCCGTGGGTTTTCCATCCAATGTCGTATTACCACTGAAGATCCCTCTAAGAATTTC
CAACCGGATACCGGTCGCCTGGAGGTCTATCGTTCTGCCGGTGGTAATGGTGTGAGATTGGA
CGGTGGTAACGCTTATGCAGGTGCTACTATCTCGCCTCACTACGACTCAATGCTGGTCAAATG
TTCATGCTCTGGTTCTACTTATGAAATCGTCCGTAGGAAGATGATTCGTGCCCTGATCGAATT
CAGAATCAGAGGTGTTAAGACCAACATTCCCTTCCTATTGACTCTTTTGACCAATCCAGTTTT
TATTGAGGGTACATACTGGACGACTTTTATTGACGACACCCCACAACTGTTCCAAATGGTATC
GTCACAAAACAGAGCGCAAAAACTGTTACACTATTTGGCAGACTTGGCAGTTAACGGTTCTT
CTATTAAGGGTCAAATTGGCTTGCCAAAACTAAAATCAAATCCAAGTGTCCCCCATTTGCAC
GATGCTCAGGGCAATGTCATCAACGTTACAAAGTCTGCACCACCATCCGGATGGAGACAAGT
GCTACTGGAAAAGGGACCATCTGAATTTGCCAAGCAAGTCAGACAGTTCAATGGTACTCTAC
TGATGGACACCACCTGGAGAGACGCTCATCAATCTCTACTTGCAACAAGAGTCAGAACCCAC
GATTTGGCTACAATCGCTCCAACAACCGCACATGCCCTTGCAGGTGCTTTCGCTTTAGAATGT
TGGGGTGGTGCTACATTCGACGTTGCAATGAGATTCTTGCATGAGGATCCATGGGAACGTCT
GAGAAAATTAAGATCTCTGGTGCCTAATATTCCATTCCAAATGTTATTACGTGGTGCCAACGG
TGTGGCTTACTCTTCATTACCTGACAATGCTATTGACCATTTTGTCAAGCAAGCCAAGGATAA
TGGTGTTGATATATTTAGAGTTTTTGATGCCTTGAATGATTTAGAACAATTAAAAGTTGGTGT
GAATGCTGTCAAGAAGGCCGGTGGTGTTGTCGAAGCTACTGTTTGTTACTCTGGTGACATGCT
TCAGCCAGGTAAGAAATACAACTTAGACTACTACCTAGAAGTTGTTGAAAAAATAGTTCAAA
TGGGTACACATATCTTGGGTATTAAGGATATGGCAGGTACTATGAAACCGGCCGCTGCCAAA
TTATTAATTGGCTCCCTAAGAACCAGATATCCGGATTTACCAATTCATGTTCACAGTCATGAC
TCCGCAGGTACTGCTGTTGCGTCTATGACTGCATGTGCCCTAGCAGGTGCTGATGTTGTCGAT
GTAGCTATCAATTCAATGTCGGGCTTAACTTCCCAACCATCAATTAATGCACTGTTGGCTTCA
TTAGAAGGTAACATTGATACTGGGATTAACGTTGAGCATGTTCGTGAATTAGATGCATACTG
GGCCGAAATGAGACTGTTGTATTCTTGTTTCGAGGCCGACTTGAAGGGACCAGATCCAGAAG
TTTACCAACATGAAATCCCAGGTGGTCAATTGACTAACTTGTTATTCCAAGCTCAACAACTGG
GTCTTGGTGAACAATGGGCTGAAACTAAAAGAGCTTACAGAGAAGCCAATTACCTACTGGGA
GATATTGTTAAAGTTACCCCAACTTCTAAGGTTGTCGGTGATTTAGCTCAATTCATGGTTTCT
AACAAACTGACTTCCGACGATATTAGACGTTTAGCTAATTCTTTGGACTTTCCTGACTCTGTT
ATGGACTTTTTTGAAGGTTTAATTGGTCAACCATACGGTGGGTTCCCAGAACCATTAAGATCT
GATGTATTGAGAAACAAGAGAAGAAAGTTGACGTGCCGTCCAGGTTTAGAATTAGAACCATT
TGATCTCGAAAAAATTAGAGAAGACTTGCAGAACAGATTCGGTGATATTGATGAATGCGATG
TTGCTTCTTACAATATGTATCCAAGGGTCTATGAAGATTTCCAAAAGATCAGAGAAACATAC
GGTGATTTATCAGTTCTACCAACCAAAAATTTCCTAGCACCAGCAGAACCTGATGAAGAAAT
CGAAGTCACCATCGAACAAGGTAAGACTTTGATTATCAAATTGCAAGCTGTTGGTGACTTAA
ATAAGAAAACTGGGCAAAGAGAAGTGTATTTTGAATTGAACGGTGAATTAAGAAAGATCAG
AGTTGCAGACAAGTCACAAAACATACAATCTGTTGCTAAACCAAAGGCTGATGTCCACGATA
CTCACCAAATCGGTGCACCAATGGCTGGTGTTATCATAGAAGTTAAAGTACATAAAGGGTCT
TTGGTGAAAAAGGGCGAATCGATTGCTGTTTTGAGTGCCATGAAAATGGAAATGGTTGTCTC
TTCACCAGCAGATGGTCAAGTTAAAGACGTTTTCATTAAGGATGGTGAAAGTGTTGACGCAT
CAGATTTGTTGGTTGTCCTAGAAGAAGAAACCCTACCCCCATCCCAAAAAAAGTAAATTGAA
TTGAATTGAAATCGATAGATCAATTTTTTTCTTTTCTCTTTCCCCATCCTTTACGCTAAAATAA

TAGTTTATTTTATTTTTTGAATATTTTTTATTTATATACGTATATATAGACTATTATTTATCTTT
TAATGATTATTAAGATTTTTATTAAAAAAAAATTCGCTCCTCTTTTAATGCCTTTATGCAGTTT
TTTTTTCCCATTCGATATTTCTATGTATAAGTTATTAGGTCTAGAGATCCCAATACAACAGAT
CACGTGATCTTTTGTAAGATGAAGTTGAAGTGAGTGTTGCACCGTGCCAATGCAGGTGGCTA
TTAGATTAAATATGTGATTTGTTCTATTAAGTTTCCTGTATAATTACAAATGAATAACGAAAT
GAGACAAAGAAGAGAACCAAATTTTACAAGCAGCCAAGATTTCCTTGACAGCCTTGGCGATA
GCATCGCCAACCTCAGTGGTAGAGTTAGAACCACCAAGGTCACCGGTTCTGACACCTGCATC
CAAGACATTTCTAACAGCTTCTTCAAGAGCCCTACCTTCTTCAACCAAATCCAAGGATAACTT
CAACATCATAGCTGCAGATAAGATGGTAGCAATTGGGTTAACCTTGTTTGCTGGTAAATCTG
GGGCAGAACCATGACATGGTTCGTACAAACCGAATGCCTTGTTAGTGTCAGGTAGGGAAGCT
AGAGATGCAGAAGGTAATAAACCCAAAGAACCTGGAATAACAGAGGCTTCATCGGAGATAA
TATCACCAAACATGTTGTTGGTAATAACAACACCGTTTAGCTTAGTTGGTGATTTAACCAAAA
TCATAGCAGCAGAGTCGATCAATTGGTGCTGAACAGTTAATTGTGGGAACTCAGTCTTGATG
GTTTCTTCAACAGTCTTTCTCCACAATCTGGAAGAGGCAAGCACGTTAGCCTTGTCAAGTGAC
CAGATTGGTAATGGTGGGTTTTGTTGCAATGCCAAGAAAGCAGCCATTCTTGTAATTCTTTGA
ACTTCAGGAACACTGTATTTCTCAGAGTCCCAAGCAACTCCGTCACCTTCATCTTCTTTTCTTT
CACCAAAGTA

GATACCACCAACCAATTCTCTAACGACGACGAAATCGGTACCCTTTGCATATTCAGGCTTCA
AAGGAGAAAGATCTAGTAAAGAATCAGAAGCAAAGTTACATGGTCTTAAGTTGGCGTATAG
ACCCAATTCCTTTCTGATCTTCAATAGACCTTGTTCTGGTCTAACTGCGCCCGTACCCCATTTT
GGACCACCAACAGCACCTAGTAAGACAGCATCGGCTTTCTTAGAGGCTTCTAGAGCTTCATC
TGGTAAAGGAGTGCCAGTGGCATCGATGGCAGCACCCCCGATCAAGTGATGTTGGAAATTGA
ACTTAATTTCTGGACGGACTTCAGCAATGGCATTCAAGACCTTAATAGCTTCGTCAGTAACTT
CTTTACCGACGTGATCACCCGGTAGGACAACGATATTCTTAGACATGGTATAATCTGTGTAGT
GTGGGATACTTTTTACTTCTTCAAATAGGTATCAACAATAAAAATAAATCGAAGCAAATGTA
GGAATGCGTTAAAGCAGATGTACTTACTTAGAGTACATAGATATATTTATATAATTCAATATA
TAAAAGTATATGAAGCATCTGATGTTGAACCTGTCATGACTCTAAACTGGAATGGGTAGTTA
TGCTTCTGAAGGTTTTCCGTTAGAAAATGAGTGCAGGGATCAGGCCCGGAACCGGTTTTAGC
CTGAAAGGAAAAGAAAAAAATGCGGTGATTACTAGCACGTGACTGCGCTGAATTGGAATCC
ACCACAAGTACAGCGAATGGCAAGAGGGAAGGGGAATATATTGTGCCTGGCGGTAGTCTGC
TATATCAAATGTTAAACATAACTAAATGCAGTAGTGAATAGAGTAATGTATTTCTACATATGT
GGGTAGGGCCAATGGGAGCCCGATGTCTATTGTACAGATATTTTCCTTTTATAGTTAATCAAT
TCAACTTGGAAATCATTGAGTCGATATCTACTTGGATAACTGGAGCCGTGGCAGCGTTTGAA
ATTTGACGAGTCTCCTTGTTTTGTTCGCGCATTTTCAGTTTATTCAAACGGGAGATGGCACCG
AATCCACTGACTTCTCTGTTCAATTTGAATGGTAATAGGAAGAAAGTACATTTCGAGAAATTT
TCGCCCAACGTCGAAATCTCATGGATGATATCCTCGATACAAATGACACCTTCATCACCTAAT
TTCTCTTCAACGATATTGTTGTCATTCAAGATTATCTCTTTTGGTTCTTTATCTTCTGGCCTTTG
CCACATTATTCTAGATCTCTTTTGGATTAAAGAACGGATAGATGCAAGTGAAGGTTGTCCGAT
AACAATATATGGTGATACAAGTTTCAACAATGGGTACACGTCCTTGGTAAGTTTGATGAAAA
CACCAGTGTTCAACTCAACAAGTCTCAAAAGAGATAACACTTTGTAAGCCTTTGCTGGGATC
TTCACAGCGAGCTCTCGAGAACCCTTAATATAACTTCGTATAATGTATGCTATACGAAGTTAT
TAGGTGATATCAGATCCACTAGTGGCCTATGCACCCAATTCGCCCTATAGTGAGTCGTATTAC
GCGCGCTCACTGGCCGTCGTTTTACAACGTCGTGACTGGGAAAACCCTGGCGTTACCCCTGC
AGGACAGACGTCAT**TAGTGCTGAGGCATTAATTGATCATAAAACGGAATGATGAATAAT
ATTTATAGAATTGTGTAGAATTGCAGATTCCCTTTTATGGATTCCTAAATCCTCGAGGA**

GAACTTCTAGTATATTCTGTATACCTAATATTATAGCCTTTATCAACAATGGAATCCCAA
CAATTATCTCAAAATTCACATATTTCTCAAGATCTGCGGCCG
